# A Transparent Rule-Based Decision-Support Prototype for Diabetic Menu Planning and Meal-Combination Optimization: Development and Computational Evaluation

**DOI:** 10.3390/nu18193140

**Published:** 2026-09-23

**Authors:** Mirela Marić, Vesna Antoska Knights, Dardan Bytyqi, Vezirka Jankuloska, Jasenka Gajdoš Kljusurić

**Affiliations:** 1 Nutricionističko Savjetovalište za Prehranu dr. sc. Mirela Marić, Petrova 47, 10000 Zagreb, Croatia; info@mirela-maric.com; 2 Faculty of Technology and Technical Sciences, University “St. Kliment Ohridski”—Bitola, Dimitar Vlahov 57, 1400 Veles, North Macedonia; vesna.knights@uklo.edu.mk (V.A.K.); dardan.bitici@uklo.edu.mk (D.B.); vezirka.jankuloska@uklo.edu.mk (V.J.); 3 Faculty of Food Technology and Biotechnology, University of Zagreb, Pierottijeva 6, 10000 Zagreb, Croatia

**Keywords:** diabetes mellitus, medical nutrition therapy, clinical nutrition, decision support systems, rule-based systems, menu planning, mixed-integer linear programming, diet optimization

## Abstract

**Background/Objectives:** Translating medical nutrition therapy guidance into practical meal plans requires consistent application of clinical and dietetic rules while accommodating different therapeutic regimens. This study developed and computationally evaluated a transparent Python-based decision-support prototype for diabetic menu planning. **Methods:** The prototype combines procedural decision rules, food-exchange patterns, nutritional checks, and mixed-integer linear programming (MILP). Two meal-frequency pathways were represented: a conventional multi-meal pathway and a three-main-meal pathway for people treated with modern basal–bolus insulin regimens. For the multi-meal pathway, a structured dataset containing 42 candidate meals (seven alternatives in each of six meal categories) was used for objective-specific optimization and exhaustive evaluation of all 117,649 possible daily combinations under predefined nutritional constraints. Sensitivity to the allowable energy deviation and trade-offs among nutritional objectives were also examined. **Results:** The prototype produced deterministic pathway selection, exchange allocation, nutritional summaries, and explicit warnings when required information was unavailable. Under the baseline ±6.5% energy tolerance, 5628 of 117,649 six-meal combinations (4.78%) satisfied all nutritional constraints. Objective-specific optimization identified feasible solutions for minimizing energy, carbohydrate, and saturated fat and maximizing dietary fiber. The proportion of feasible combinations ranged from 2.71% at ±5% energy tolerance to 9.47% at ±10%, while comparison of objective-specific solutions demonstrated measurable nutritional trade-offs. The three-meal basal–bolus pathway generated predefined exchange allocation and clinical-review warnings but was not subjected to the same exhaustive combinatorial optimization. **Conclusions:** The prototype demonstrates the computational feasibility of integrating explicit dietetic rules, regimen-related meal-pattern selection, food-exchange allocation, and mathematical optimization within an inspectable decision-support framework. The findings establish technical feasibility within the evaluated datasets rather than clinical effectiveness. Independent software testing, expert usability assessment, evaluation using larger and version-controlled meal datasets, and prospective clinical validation are required before routine clinical implementation.

## 1. Introduction

Diabetes mellitus (DM) represents one of the most significant global public health challenges of the 21st century [1,2,3,4,5]. Current epidemiological estimates indicate that more than 537 million adults worldwide are living with diabetes [6], with prevalence continuing to increase across all regions and age groups. Some studies predict that type 2 diabetes will affect approximately 853 million adults by 2050 if current trends continue [7]. Type 2 diabetes accounts for the majority of cases and is strongly associated with lifestyle-related factors, including sedentary behavior, suboptimal dietary patterns, and increasing rates of overweight and obesity [5,8]. Beyond impaired glycemic regulation, diabetes is associated with a broad spectrum of microvascular and macrovascular complications that substantially reduce quality of life, increase morbidity and mortality, and impose a considerable economic burden on healthcare systems [7,9,10].

Achieving and maintaining adequate glycemic control is essential for preventing or delaying diabetes-related complications. Medical nutrition therapy (MNT) represents a fundamental component of diabetes management and has demonstrated significant effects on glycemic control, body weight regulation, lipid metabolism, and cardiovascular risk reduction [5,7]. Evidence supports the role of individualized dietary interventions tailored to patients’ metabolic goals, clinical characteristics, dietary preferences, and cultural context [11]. Structured meal planning and nutrition education can lead to clinically meaningful improvements [12] in glycemic markers, including reductions in glycated hemoglobin (HbA1c) in individuals with type 2 diabetes.

Despite the established effectiveness of nutritional interventions, their implementation in routine clinical practice remains challenging. Long-term adherence to dietary recommendations is frequently limited by the complexity of daily meal planning, individual variability, comorbidities, and the need for continuous adjustment of nutritional strategies [8,13]. Glycemic variability is influenced not only by the total intake of nutrients but also by factors such as meal timing, carbohydrate distribution, and overall dietary composition [1]. Therefore, effective diabetes nutrition management requires approaches that combine evidence-based recommendations with practical tools capable of supporting individualized and sustainable dietary planning [5,7,8,14].

In Croatia, national guidelines for medical nutrition therapy in adults with diabetes were updated in 2023 and include recommendations on energy intake, macronutrient distribution, carbohydrate quality, fiber, sodium, and meal frequency [15]. Importantly, meal frequency is related to the therapeutic regimen. A structured five- or six-meal pattern may be used with lifestyle treatment, oral glucose-lowering therapy, basal insulin, or selected conventional insulin regimens, whereas three principal meals without routine snacks may be appropriate for people using modern basal–bolus therapy or multiple daily injections, provided that meal timing and carbohydrate intake are coordinated with insulin action.

Designing diabetic menus is inherently complex, requiring simultaneous consideration of multiple factors, including individual energy requirements, macronutrient distribution, carbohydrate allocation, food composition, dietary restrictions, comorbidities, and patient-specific nutritional needs [11,16,17,18]. In clinical environments characterized by high patient workload and limited resources, maintaining consistency with evidence-based recommendations can be challenging [18]. This highlights the need for digital tools capable of assisting healthcare professionals by automating routine aspects of nutritional planning while preserving clinical accuracy and flexibility [8,19].

Computational and decision-support technologies in clinical nutrition provide opportunities for improving personalized dietary management. Rather than replacing clinical expertise [20], decision-support systems can support healthcare professionals by integrating nutritional rules, patient characteristics, and evidence-based recommendations into structured decision-making processes [21,22]. Rule-based systems based on predefined knowledge and explicit decision logic may facilitate standardized, reproducible, and transparent decision support. However, their application to automated diabetic menu decision remains relatively limited, particularly when considering multiple energy levels, dietary modalities, and specific clinical requirements [21]. Developing such systems requires the formalization of nutritional guidelines into computational rules that can generate clinically appropriate meal plans while maintaining adaptability to individual patient needs [5,7,8]. Therefore, the aim of this study was to develop a transparent rule-based decision-support prototype that translates selected recommendations from the 2023 Croatian guidelines into meal-pattern selection, exchange allocation, nutritional validation, and menu-combination optimization. The prototype distinguishes a conventional six-meal pattern from a three-meal basal–bolus pattern and evaluates the feasibility of combining candidate meals under predefined nutritional constraints. The methodological contribution of the study lies not in introducing rule-based decision support or mathematical diet optimization as independent methods but in integrating explicit guideline-derived procedural rules, therapy-related meal-pattern selection, food-exchange allocation, nutrient-level feasibility constraints, mathematical optimization, and traceable decision outputs within a single computational framework. In addition, exhaustive enumeration of the defined multi-meal candidate space enables the prototype to characterize not only individual optimized menus but also the proportion and nutritional properties of combinations satisfying the implemented constraints.

## 2. Materials and Methods

### 2.1. Study Design

This study describes the development of a Python 3.14-based decision-support prototype for adult diabetic menu planning. The computational framework comprised two complementary components: (i) procedural decision rules for patient-input validation, therapy-based selection of a conventional multi-meal or three-meal basal–bolus pathway, exchange allocation, and warning generation and (ii) mixed-integer optimization of a structured six-meal candidate dataset. The rules were informed primarily by the 2023 Croatian guidelines for medical nutrition therapy in diabetes. Figure 1 and Figure 2 present the conceptual architecture and implemented computational workflow, respectively.

The current prototype is intended as a professional decision-support tool for use by dietitians/nutritionists and other appropriately qualified healthcare professionals involved in diabetes nutrition care. It is not intended for autonomous patient use or to replace individualized clinical and dietetic judgment.

### 2.2. Knowledge Base Development

The development followed a knowledge engineering framework to translate clinical guidelines into executable decision logic. Primary knowledge sources incorporated into the system are summarized in Table 1.

#### 2.2.1. Source Guidelines and Rule Extraction

Guideline recommendations were extracted and categorized into functional rule sets: (i) Target Statement Identification; (ii) Rule Atomization; and (iii) Module Categorization. This refers to identified clinical statements regarding caloric targets, macronutrient ranges, glycemic control, and meal frequency; the reduction of narrative statements to individual, atomic logical conditions to maintain system traceability; and the rules organized into functional modules (Energy, Macronutrients, Meal Frequency, Diet Type, Serving/Exchange, Safety/Comorbidities, and Exercise interaction). Rule extraction and computational encoding were performed within the multidisciplinary author team, which included three nutrition professionals, two based in Croatia and one in North Macedonia, together with two engineering/technology researchers involved in computational implementation. One of the Croatian nutrition professionals is engaged in clinical nutrition practice, while another has additional expertise in nutritional modeling and optimization. Clinical recommendations were first interpreted from a nutrition-care perspective and subsequently translated into explicit computational conditions and quantitative constraints, with interdisciplinary review of their computational implementation. Where a source recommendation did not permit an unambiguous computational interpretation, it was not treated as an independently validated clinical rule; instead, the corresponding assumption or operational modeling decision was made explicit in the prototype or retained as requiring professional assessment. The resulting rule set underwent internal multidisciplinary review within the author team, but no formal independent external validation by a separate panel of diabetes-specialist dietitians or physicians was performed at this developmental stage.

#### 2.2.2. Clinical Rules and Nutritional Constraints

The embedded clinical logic follows these constraints:

Energy Requirements: Adjusted using guideline equations according to BMI, age, and physical activity [15].

Macronutrient Ranges: Carbohydrates (40–55% energy), protein (a minimum baseline intake of 0.8 g/kg body weight/day, with individualized adjustment in diabetic kidney disease), and total fats (20–35% energy) were derived from source guidelines [15].

Carbohydrate (CHO) Quality & Fiber: The source guidelines emphasize carbohydrate quality and fiber-rich foods, including a fiber density of at least 14 g/1000 kcal and evidence supporting daily fiber intakes of approximately 25–29 g [15]. For the computational optimization scenario, a minimum fiber threshold of 25 g/day was operationally selected.

Sodium and Saturated Fat: Sodium intake was limited to ≤2300 mg/day in accordance with the source guidelines [15]. The guidelines recommend limiting saturated-fat intake without defining a single universal gram-based threshold; therefore, the ≤14 g/day saturated-fat boundary used in the optimization model represents an operational modeling threshold for the evaluated computational scenario [15].

Meal Distribution: Meal frequency was implemented according to therapy-related recommendations in the source guidelines. The conventional multi-meal pathway comprises three main meals and planned snacks, resulting in five or six eating occasions depending on the prescribed pattern. The basal–bolus pathway comprises three principal meals without routine snacks, subject to individualized assessment of insulin timing and hypoglycemia risk [15].

The distinction between guideline-derived recommendations and computational operationalization was maintained during model development. Where the source guidance provided a range or required individualized clinical interpretation, fixed numerical boundaries required for deterministic computation were treated as modeling decisions for the evaluated scenarios rather than as universally applicable clinical prescriptions.

#### 2.2.3. Serving and Exchange System

To operationalize energy and macronutrient targets, the prototype incorporates standardized clinical exchange patterns across the prescribed energy tiers. In the conventional pathway, carbohydrate, protein, and fat exchanges are distributed across five or six eating occasions, depending on the selected pattern. In the basal–bolus pathway, exchanges are distributed across three principal meals. Daily carbohydrate, protein, and fat exchange servings are systematically distributed across meals to ensure glycemic stability and support insulin-treated regimens. The detailed target exchange allocations and meal-by-meal serving distribution schemes across all energy levels are provided in Supplementary Materials (Tables S1 and S2). The operational translation relies on Croatian dietetic exchange units across energy levels (1200–2200 kcal), where 1 CHO Serving averages 15 g carbohydrates, 1 Protein Serving averages 7 g protein, 1 Fat Serving averages 5 g fat and Dairy/Milk Serving averages 12 g CHO and 8 g protein [23].

### 2.3. Rule Base Construction and System Architecture

The rules were implemented as explicit Python conditions and functions. The current prototype does not use CLIPS syntax or a separate production-rule engine; consequently, the term rule-based refers to transparent, predefined conditional logic rather than a CLIPS-compatible forward-chaining implementation.

$$\left.IF\;\left(condition\left(s\right)\right)\;\right\rangle \;\;\;THEN\;(actions\left(s\right))$$

Patient inputs are evaluated sequentially against predefined conditions. These conditions determine the meal-frequency pathway, relevant comorbidity warnings, exchange allocation, optimization objective, and output. Representative modules are summarized in Table 2.

#### 2.3.1. Interactive Patient Input and Inference Interface

To operationalize the rule-based architecture, an interactive computational interface was implemented in Python v. 3.14. within the Jupyter environment. The interface enables structured entry of patient-specific and dietary parameters, including age, sex, body weight, height, diabetes type, therapy, insulin-delivery modality, hypertension, chronic kidney disease, physical activity, nutritional goal, target energy level, and optimization objective.

Input data are first subjected to plausibility and consistency checks. Derived parameters, including body mass index (BMI), are then calculated. Therapy information is used to select either the conventional five- or six-meal optimization pathway or the three-meal basal–bolus exchange pathway. Branch selection is based on the insulin regimen and required meal structure, not on the physical insulin-delivery device alone.

The interface therefore represents the operational entry point of the decision-support prototype, linking patient data acquisition with rule activation, inference, menu generation, nutritional validation, and personalized recommendations. A representative view of the interface is provided in Figure A1.

#### 2.3.2. Explainability and Decision Traceability

Explainability was implemented through a printed decision trace. For each evaluated profile, the prototype reports the selected pathway, triggered conditions, optimization objective or exchange pattern, available validation results, warnings, and resulting recommendations.

The trace provides an auditable link between entered variables and output. In particular, a modern basal–bolus regimen activates the three-meal pathway; BMI and comorbidities add contextual recommendations or warnings. Use of a pen injector alone should not be interpreted as sufficient clinical justification for this pathway.

The system additionally distinguishes between parameters that can be computationally validated and parameters for which the available dataset is insufficient. In such cases, the system generates an explicit clinical-review warning rather than an unsupported PASS decision. In addition to the representative patient-specific example presented in the Results, additional decision-trace examples covering different patient characteristics, computational pathways, and comorbidity scenarios are provided in Appendix A (Table A2, Table A3 and Table A4). These examples illustrate how differences in therapy, BMI, and the presence of hypertension or chronic kidney disease affect pathway selection, rule activation, clinical-review warnings, and the resulting recommendations.

### 2.4. Food Composition Database

A relational database was constructed using standard national and clinical sources:

Data Sources: Primary data were derived from official Food Composition Tables, standard hospital menu manuals, and verified clinical nutrition software records [24,25,26].

Exchange Mapping: Each food entry was assigned standard exchange values (CHO, protein, fat units) per standard portion size [26] to enable direct inference matching by the rule engine.

#### 2.4.1. Computational Meal Dataset Preparation

For computational menu selection, the nutritional data were reorganized into a structured meal-level dataset. Seven alternative daily menus were decomposed into six meal categories: breakfast, morning snack, lunch, afternoon snack, dinner, and night snack, yielding 42 candidate meals (seven alternatives per meal category). The candidate meals were derived from actual hospital menu offerings classified as diabetic diets and previously used in the analysis of hospital diet offers reported by Resman et al. [26]. The present computational dataset was constructed from seven daily diabetic menu offerings, providing seven alternatives within each of the six meal categories and resulting in 42 candidate meals. Thus, the dataset reflects meals used in a real hospital dietary context rather than synthetically generated meal options. However, the seven daily menus were not selected through a formal sampling procedure designed to establish representativeness of the broader range of diabetic diets or hospital food services. Accordingly, they should be regarded as a clinically grounded but restricted candidate dataset for computational evaluation of the prototype. Each candidate meal was represented by its energy content, protein, total fat, carbohydrates, saturated fatty acids (SFAs), dietary fiber, and sodium. Comprehensive micronutrient variables were not included in the computational meal dataset; therefore, the prototype does not perform automated micronutrient verification, and nutritional validation is limited to the variables represented in the dataset.

The dataset was programmatically preprocessed to standardize meal identifiers, remove duplicate records, assign candidates to their corresponding meal categories, convert nutritional variables to numerical format, and verify data completeness. The resulting 42-meal matrix served as the computational input for subsequent menu optimization and nutritional constraint validation.

#### 2.4.2. Mathematical Optimization Formulation

The menu-selection problem was formulated as a binary mixed-integer linear programming (MILP) problem. Each candidate meal was represented by a binary decision variable indicating whether the meal was selected. Exactly one candidate meal was selected from each of the six meal categories—breakfast, morning snack, lunch, afternoon snack, dinner, and night snack—subject to the predefined nutritional constraints.

Let $x_{i}$ denote the binary decision variable for candidate meal i:

(1) $$x_{i}=f\left(x\right)=\left\{\begin{array}{c} 1,\;\;\;\;if\;candidate\;meal\;i\;is\;selected\\ 0,\;\;\;\;otherwise \end{array}\right.$$

For each meal category $g_{i}$, exactly one candidate meal was selected:

(2) $$\sum_{i\in G_{g}}x_{i}=1,g=1,\ldots ,6$$

where $G_{i}$ denotes the set of candidate meals belonging to meal category g.

Total daily energy, protein, fat, carbohydrates, saturated fatty acids (SFAs), dietary fiber, and sodium were calculated as weighted sums of the nutritional composition of the selected meals. For a nutritional variable *a_i_*, the corresponding daily total *A* was calculated as:

(3) $$A=\sum_{i=1}^{42}a_{i}x_{i}$$

where *a_i_* represents the amount of the respective nutritional variable in candidate meal *i*, and 42 is the total number of candidate meals.

For the 1800-kcal reference scenario, feasibility was defined by an energy range of 1683–1917 kcal/day (±6.5% of the target), total fat of 40–70 g/day, carbohydrates of 180–247.5 g/day, protein ≥64 g/day, SFA ≤14 g/day, dietary fiber ≥25 g/day, and sodium ≤2300 mg/day.

Protein was included as a baseline adequacy constraint in the conventional multi-meal optimization pathway rather than as a universally fixed target. The implemented optimization does not automatically apply a CKD-stage-specific protein prescription. When chronic kidney disease is identified, the prototype therefore flags the case for clinical review rather than treating satisfaction of the general protein constraint as confirmation of renal nutritional adequacy. Individualized protein prescription in CKD requires consideration of kidney-disease stage and other patient-specific clinical factors and remains outside the automated optimization scope of the current prototype [15]. These fixed gram-based boundaries operationalize the broader guideline-derived recommendations for the evaluated 1800-kcal computational scenario and should not be interpreted as universally applicable individualized clinical prescriptions. The ±6.5% energy tolerance and the fixed saturated-fat boundary were modeling parameters used to define the baseline computational scenario rather than direct guideline prescriptions. The ±6.5% tolerance was selected as the baseline modeling scenario to permit limited deviation around the nominal 1800-kcal target while retaining sufficient selectivity for evaluation of the candidate space; because this tolerance was not derived as a clinical prescription, additional ±5% and ±10% scenarios were evaluated to assess the sensitivity of feasibility results to this modeling choice. Four separate objective functions were evaluated.

Minimization of total energy:

(4) $$\min Z_{E}=\sum_{i=1}^{42}E_{i}x_{i}$$

Minimization of carbohydrate intake:

(5) $$\min Z_{C}=\sum_{i=1}^{42}C_{i}x_{i}$$

Minimization of saturated fatty acids:

(6) $$\min Z_{SFA}=\sum_{i=1}^{42}{SFA}_{i}x_{i}$$

Maximization of dietary fiber:

(7) $$\max Z_{Fib}=\sum_{i=1}^{42}{Fib}_{i}x_{i}$$

The four objective functions were selected as contrasting computational scenarios to demonstrate how the optimization framework selects among meal combinations that already satisfy the predefined feasibility constraints. They should not be interpreted as indicating that lower energy, carbohydrate, or saturated-fat intake, nor higher fiber intake, is inherently preferable for every individual with diabetes. In particular, the minimum-energy and minimum-carbohydrate objectives were used to examine the lower boundaries attainable within the feasible candidate space rather than to recommend unrestricted reduction of energy or carbohydrate intake. Selection of an optimization objective for clinical application would require individualized professional assessment. Computational analyses were implemented in Python within a Jupyter Notebook environment. In the available prototype, mixed-integer optimization is formulated with PuLP and solved as a binary linear-programming problem. The optimization model was solved using the solver invoked by PuLP through the default model.solve() call. No user-defined time limit, relative or absolute optimality-gap threshold, thread count, or other solver-specific parameter was specified. Solver termination status was retrieved through LpStatus[model.status], and an optimization result was accepted only when the returned status was “Optimal”; all other termination statuses were treated as unsuccessful optimization outcomes.

#### 2.4.3. Exhaustive Feasibility, Sensitivity, and Trade-Off Analysis

To complement mathematical optimization, an exhaustive combinatorial analysis was used to characterize the candidate solution space. With seven alternatives in each of six meal categories, selection of one candidate per category yields 7^6^ = 117,649 possible daily combinations. Each combination was evaluated against the constraints defined in Section 2.4.2.

To assess the sensitivity of the feasible solution space to the allowable deviation from the 1800-kcal reference target, the analysis was repeated using energy tolerances of ±5%, ±6.5%, and ±10%, while all other nutritional constraints were held constant. The 1800-kcal level was selected as a reference computational scenario for detailed combinatorial analysis because it represents an intermediate energy level within the range implemented in the prototype and provides a single standardized scenario in which the behavior of the optimization framework and the effect of alternative energy tolerances could be examined. The exhaustive optimization and sensitivity results reported in this study therefore characterize this reference scenario and should not be interpreted as demonstrating equivalent optimization performance across all implemented energy levels. For each tolerance level, the number and proportion of feasible combinations were calculated, and the optimal values for minimum energy, minimum carbohydrate intake, minimum SFA intake, and maximum dietary fiber were determined.

Finally, objective trade-offs were assessed using the optimal solutions obtained under the baseline ±6.5% energy tolerance. Relative percentage differences between selected objective-specific solutions were calculated to quantify the nutritional consequences of prioritizing one optimization objective over another.

### 2.5. Execution and Validation Workflow

The prototype operates as a transparent decision-support workflow. It collects patient and therapy information, performs input plausibility checks, calculates body mass index (BMI), evaluates relevant clinical conditions, and selects the applicable meal-pattern pathway. Depending on the therapeutic regimen and selected inputs, the program proceeds either to the conventional multi-meal optimization pathway or to the three-meal basal–bolus exchange pathway.

Within the conventional multi-meal pathway, the evaluated optimization dataset comprised 42 candidate meals organized into six meal categories, with seven alternatives in each category. The optimization component used PuLP to select one candidate from each category while applying the predefined energy and nutritional constraints. The use of six categories describes the structure of the evaluated optimization dataset and does not imply that every conventional dietary pattern necessarily comprises six eating occasions.

The user selects the optimization objective: minimization of total energy, carbohydrate intake, or saturated fatty acids, or maximization of dietary fiber. If a feasible combination satisfying all implemented constraints is identified, the prototype reports the optimized menu combination and its nutritional summary. If no feasible combination is identified, the program reports that no feasible solution was found; it does not automatically modify recipes or generate replacement foods.

Within the three-meal basal–bolus pathway, the prototype retrieves a predefined energy-specific exchange pattern comprising breakfast, lunch, and dinner. It subsequently reports the reference energy value, daily macronutrient totals, and meal-level carbohydrate allocation. This pathway does not use the 42-meal candidate dataset or PuLP optimization. Accordingly, exhaustive combinatorial optimization was not performed for the three-meal basal–bolus pathway. Technically, the current implementation retrieves predefined energy-specific exchange patterns rather than selecting among a structured meal-level candidate library; therefore, it does not define a combinatorial candidate space equivalent to the 42-meal multi-meal dataset. Clinically, meal planning in the basal–bolus pathway requires individualized consideration of insulin timing and hypoglycemia risk. Applying the same static meal-combination optimization used for the conventional pathway would therefore not represent an equivalent evaluation of the two pathways. Development of a dedicated three-meal candidate-meal dataset and incorporation of the relevant patient-specific insulin-related parameters would be required before a comparable optimization analysis could be appropriately evaluated. Nutritional checks are limited to variables represented in the corresponding dataset. When clinically relevant nutrient information is unavailable in the three-meal exchange dataset, the prototype generates a clinical-review warning rather than an unconditional compliance decision. For patients with CKD, the resulting clinical-review warning also indicates that protein requirements require individualized professional assessment and are not automatically validated by the general computational constraints. Outputs from both pathways include a decision trace and require interpretation and, where appropriate, adaptation by a qualified dietitian/nutritionist.

Figure 2 summarizes the implemented sequence from patient input to pathway selection, calculation, validation, and output. The mathematical optimization and the three-meal exchange pathway are parallel components; only the structured conventional multi-meal candidate dataset was used for the exhaustive feasibility and sensitivity analyses.

### 2.6. Technical and Operational Verification

Operational evaluation focused on whether identical inputs produced the same pathway, calculations, and printed warnings. This deterministic behavior demonstrates technical consistency of the prototype but does not constitute clinical validation, diagnostic validation, or evidence of patient benefit.

The rule-based architecture enabled transparent decision-making, where every generated recommendation could be traced to predefined clinical production rules derived from the Croatian medical nutrition therapy guidelines. The validation mechanism ensured that candidate menus violating the predefined nutritional constraints were identified as infeasible and excluded from the feasible solution set, while cases without a feasible solution were explicitly reported rather than automatically modified or regenerated. Together, these characteristics support the technical consistency, traceability, and explainability of the prototype within the tested scenarios. The principal operational characteristics are summarized in Table A1.

## 3. Results

### 3.1. Preliminary Technical Verification and Menu Generation Overview

Technical and operational checking at this developmental stage constituted internal preliminary verification rather than formal independent software verification. In the tested examples, the prototype executed the implemented input checks, pathway-selection conditions, exchange calculations, and optimization routines without runtime conflicts, and the conventional multi-meal and three-meal basal–bolus pathways produced different meal structures consistent with their encoded exchange schemes. A formal software-testing protocol comprising documented unit tests, systematic boundary-condition and invalid-input test suites, and independent recalculation by a separate tester was not performed. Consequently, these findings should be interpreted as preliminary evidence of internal computational consistency rather than as independent validation of software correctness.

Nutritional assessment depended on the variables available in each data source. The structured conventional multi-meal dataset supported evaluation of energy, macronutrients, saturated fat, fiber, and sodium. The three-meal exchange tables supported energy and macronutrient allocation but required explicit professional review for nutrients not represented in the tables. A structured summary of the representative scenarios used to illustrate variation in diabetes type, treatment regimen, BMI category, hypertension, chronic kidney disease, energy level, and computational pathway is provided in Appendix A (Table A5). This scenario matrix is descriptive and should not be interpreted as a formal software-validation test suite.

### 3.2. Compliance with Predefined Nutritional ConstraintsAcross Energy Levels

For the selected menu sets, energy and macronutrient distributions were compared with the predefined analysis thresholds. These thresholds were used as computational feasibility criteria and should not be interpreted as proof of clinical efficacy or individualized therapeutic appropriateness.

The two pathways differ primarily in meal frequency and carbohydrate timing. The conventional pathway distributes intake across six eating occasions, whereas the basal–bolus pathway uses three main meals without routine snacks. Both pathways should apply the same principles of food quality and individualized medical nutrition therapy; the basal–bolus pathway does not inherently require refined foods, reduced fiber, peeled fruit, or texture modification.

### 3.3. Multi-Meal Versus Three-Meal Basal–Bolus Patterns

The prototype distinguishes the pathways according to therapeutic context and intended meal frequency. It does not impose texture or digestibility restrictions solely because a patient uses insulin. The principal differences are summarized in Table 3.

### 3.4. Illustrative Meal Structures

Table 4 illustrates the structural difference between the multi-meal and three-meal pathways. The examples are not nutritionally interchangeable prescriptions and require individual assessment by a qualified dietitian/nutritionist.

### 3.5. Validation Module Outcomes

Carbohydrate allocation across meals at the available prescribed energy levels is presented in Figure 3. Figure 4 summarizes energy, fiber, macronutrient distribution, and sodium for five selected conventional multi-meal menus from the nominal 1800-kcal candidate set. These descriptive values were calculated from the source menu worksheets and are distinct from the objective-specific 1800-kcal optimization analysis in Table 5, Table 6 and Table 7.

The average of daily menus shown in Figure 4 were within the nominal energy range used for menu selection, but they did not all satisfy every strict nutrient threshold: one provided 24.86 g fiber and two provided 2321.07 and 2349.41 mg sodium. These observations support use of explicit warning flags and professional review rather than an unconditional claim of complete compliance.

### 3.6. Optimization and Constraint Feasibility

To evaluate optimization and constraint feasibility, the structured dataset of 42 candidate meals was subjected to objective-specific optimization. The optimization model selected exactly one meal from each of six meal categories—breakfast, morning snack, lunch, afternoon snack, dinner, and night snack—while simultaneously enforcing the predefined nutritional constraints. Four primary optimization objectives were evaluated: minimization of total energy, minimization of carbohydrate intake, minimization of saturated fatty acids (SFAs), and maximization of dietary fiber. The computational procedure used to identify feasible meal combinations and determine the optimal solution for each objective is summarized in Algorithm 1. While the decision-support prototype was designed to generate menus across six operational energy levels (1200–2200 kcal), the 1800-kcal tier was selected as an independent reference scenario for the objective-specific optimization analysis.
**Algorithm 1.** Objective-Specific Menu Optimization and Constraint-Feasibility Procedure**Input:** Structured dataset of 42 candidate meals grouped into six meal categories; 1800-kcal reference energy tier; predefined nutritional constraints.**Output:** Optimal feasible daily menu and corresponding nutritional profile for each optimization objective.Load the structured dataset of 42 candidate meals. Group candidate meals into six categories: breakfast, morning snack, lunch, afternoon snack, dinner, and night snack. Select exactly one candidate meal from each meal category. Calculate the total daily energy, protein, fat, carbohydrates, SFA, dietary fiber, and sodium of the selected multi-meal combination. Apply the predefined nutritional constraints: Reference energy tier for optimization: 1800 kcal (selected from the six system energy tiers: 1200–2200 kcal); allowable range: 1683–1917 kcal/day (±6.5%); Fat: 40–70 g/day; Carbohydrates: 180–247.5 g/day; Protein: ≥64 g/day; SFA: ≤14 g/day; Dietary fiber: ≥25 g/day; Sodium: ≤2300 mg/day. If one or more constraints are violated, reject the candidate combination. Retain combinations satisfying all nutritional constraints as feasible solutions. Independently optimize each objective: minimize total energy; minimize carbohydrate intake; minimize SFA intake; maximize dietary fiber intake. For each objective, identify the optimal feasible multi-meal combination and calculate its complete nutritional profile. Report the optimal solution, nutritional composition, and solver status for each optimization objective.**End**

For the optimization analysis, the 1800-kcal energy level was selected as the reference scenario. Consistent with the allowable energy deviation applied in the nutritional evaluation, the acceptable energy range was set at ±6.5% of the target value (1683–1917 kcal/day). The implemented constraint set further required total fat to provide 20–35% of target energy (40–70 g/day), carbohydrates to provide 40–55% of target energy (180–247.5 g/day), and protein amounts of at least 64 g/day; dietary fiber was set to be at least 25 g/day, sodium to remain at or below 2300 mg/day, and SFAs to remain at or below 14 g/day. All four optimization problems reached an optimal solver status and produced feasible daily menu combinations satisfying the implemented constraint set (Table 5).

All four optimization objectives produced feasible solutions within the predefined nutritional constraints. The minimum-energy solution yielded a total daily energy intake of 1776.97 kcal, with 86.75 g protein, 52.43 g fat, 239.52 g carbohydrates, 13.75 g SFAs, 35.50 g dietary fiber, and 1097.67 mg sodium. Minimization of carbohydrate intake resulted in the lowest carbohydrate content of 210.30 g/day at an energy intake of 1796.14 kcal. The SFA-minimization scenario achieved the lowest saturated fatty acid intake of 12.49 g/day, while the fiber-maximization scenario produced the highest dietary fiber content of 41.42 g/day at 1795.07 kcal. These findings demonstrate that different nutritional objectives resulted in distinct feasible meal combinations while satisfying all implemented nutritional constraints.

A supplementary protein-minimization analysis was performed to characterize the lower bound of protein intake achievable within the available 42-meal candidate set while retaining all other nutritional constraints. The minimum feasible protein content was 82.03 g/day, corresponding to an energy intake of 1794.57 kcal/day. This result indicates that, within the existing candidate meal database and under the implemented energy, fat, carbohydrate, SFAs, fiber, and sodium constraints, protein intake could not be reduced below 82.03 g/day. Therefore, the protein content of the optimized menus was influenced not only by the optimization objective but also by the nutritional composition of the available meal candidates.

### 3.7. Feasible Solution Space and Energy-Tolerance Sensitivity

While the objective-specific optimization identified the best feasible solution for each nutritional objective, it did not characterize the size of the overall feasible solution space. Therefore, an exhaustive combinatorial analysis was performed across all 117,649 six-meal combinations generated from the 42-meal candidate dataset (7 alternatives in each of 6 meal categories) to determine how many combinations satisfied the complete set of nutritional constraints. In addition, the robustness of feasibility and the objective-specific optima was evaluated under alternative energy tolerances of ±5%, ±6.5%, and ±10%, while all other nutritional constraints were held constant. The results are summarized in Table 6.

The exhaustive combinatorial analysis was restricted to the conventional multi-meal pathway, for which the 42-meal candidate dataset generated 117,649 possible six-meal combinations. Of these, 5628 combinations satisfied all predefined nutritional constraints under the baseline ±6.5% energy tolerance, corresponding to 4.78% of the evaluated combinatorial space. The three-meal basal–bolus pathway was not included in this combinatorial analysis because it uses predefined energy-specific exchange patterns rather than the 42-meal candidate dataset. Thus, within the conventional multi-meal pathway, the simultaneous application of the predefined nutritional constraints substantially restricted the number of feasible meal combinations. Exhaustive evaluation of all 117,649 possible six-meal combinations identified 5628 feasible daily menus under the baseline ±6.5% energy tolerance, corresponding to 4.78% of the complete combinatorial solution space.

Energy-tolerance sensitivity analysis demonstrated the expected expansion of the feasible solution space as the allowable energy range increased (Table 6). Under the more restrictive ±5% tolerance, 3192 combinations (2.71%) satisfied all constraints. At the baseline ±6.5% tolerance, this increased to 5628 combinations (4.78%), while a ±10% tolerance yielded 11,144 feasible combinations (9.47%). Thus, increasing the allowable energy deviation from ±5% to ±10% increased the number of feasible menu combinations by approximately 3.5-fold.

The objective-specific solutions also showed different degrees of sensitivity to the energy tolerance. The minimum achievable energy decreased from 1807.37 kcal at ±5% to 1776.97 kcal at ±6.5% and 1710.13 kcal at ±10%, reflecting the direct effect of relaxation of the lower energy bound. Similarly, the minimum carbohydrate intake decreased from 213.99 to 210.30 and 203.66 g/day, respectively, while the minimum achievable SFAs intake decreased from 12.52 to 12.49 and 10.39 g/day. In contrast, maximum dietary fiber increased from 39.01 g/day at ±5% to 41.42 g/day at ±6.5% and remained unchanged at ±10%, indicating that the fiber-maximizing solution had already been reached within the baseline feasible region.

### 3.8. Trade-Off Analysis Between Nutritional Objectives

Comparison of the objective-specific optimal solutions revealed clear nutritional trade-offs despite all solutions satisfying the same underlying feasibility constraints. The most pronounced contrast was observed between carbohydrate minimization and fiber maximization. The minimum-CHO solution contained 210.30 g/day of carbohydrates and 27.35 g/day of fiber, whereas the maximum-fiber solution contained 245.94 g/day of carbohydrates and 41.42 g/day of fiber. Thus, maximizing fiber increased dietary fiber by 51.4% relative to the minimum-CHO solution but was accompanied by a 16.9% increase in carbohydrate intake (Table 7).

Conversely, selecting the minimum-CHO solution reduced carbohydrate intake by 14.5% relative to the maximum-fiber solution but resulted in a 34.0% reduction in dietary fiber. A similar, although less pronounced, trade-off was observed between SFA minimization and fiber maximization. The minimum-SFA solution reduced SFA from 13.76 to 12.49 g/day (9.2%) relative to the maximum-fiber solution, while dietary fiber decreased from 41.42 to 33.33 g/day (19.5%).

These results demonstrate that no single feasible menu simultaneously optimized all nutritional objectives. Instead, the optimization framework identified distinct solutions according to the selected nutritional priority while preserving compliance with the common clinical constraint set.

### 3.9. Interactive Decision-Support Demonstration and Personalized Recommendations

To demonstrate the workflow, a representative profile was entered into the prototype: a 40-year-old man with type 1 diabetes, a modern basal–bolus insulin regimen administered by pen, body weight 100 kg, height 190 cm (BMI 27.70 kg/m^2^), hypertension, and no chronic kidney disease.

The profile activated the three-meal basal–bolus pathway, overweight-related energy review, hypertension-related sodium review, exchange allocation, selection of the 1600-kcal pattern, and three-main-meal distribution. Table 8 summarizes the resulting trace.

The generated three-meal basal–bolus exchange plan produced daily totals of 189 g carbohydrate, 78 g protein, and 38.5 g fat. These values reproduced the corresponding reference spreadsheet values for the selected 1600-kcal energy class. The calculated reference energy value was 1527.5 kcal, corresponding to a 4.5% deviation from the nominal 1600-kcal level and therefore remaining within the predefined maximum deviation of 6.5%.

Carbohydrate allocation across the three main meals was 72 g at breakfast, 55 g at lunch, and 62 g at dinner. Because hypertension was present, but sodium content was not available in the three-meal basal–bolus exchange dataset, the system did not generate an unconditional PASS decision. Instead, the final status was PASS WITH CLINICAL REVIEW REQUIRED, accompanied by a recommendation for separate sodium assessment by a qualified dietitian/nutritionist.

The recommendation module advised individualized weight-management review, use of the selected 1600-kcal exchange pattern as an illustrative planning level, preservation of meal-specific carbohydrate allocation, lower-sodium food selection, and coordination of carbohydrate intake with the prescribed insulin regimen.

After exchange allocation, the prototype displayed an illustrative three-meal menu (Table 9). The food examples demonstrate how the carbohydrate distribution could be translated into meals; they were not generated or nutritionally validated from the Croatian food-composition workbook by the current notebook.

**Table 9:** Illustrative three-meal basal–bolus menu for the evaluated profile.

Meal	Illustrative Meal (Requires Independent Nutrient Calculation)	CHO Allocation
Breakfast	Oatmeal or another measured cereal portion with milk/yogurt and fruit	72 g
Lunch	Lean protein, measured whole-grain or starchy-food portion, and vegetables	55 g
Dinner	Protein source, measured carbohydrate portion, and vegetables	62 g

The exchange allocation, rather than the illustrative food list, is the quantitative output of this pathway. Exact recipes, portions, fiber, sodium, and micronutrient content require separate calculation and professional review by a qualified dietitian/nutritionist. To complement this primary demonstration, three additional representative decision-trace scenarios are provided in Appendix A (Table A2, Table A3 and Table A4). These examples include the conventional multi-meal optimization pathway and additional basal–bolus/PEN scenarios with different BMI categories and comorbidity profiles, including chronic kidney disease and combined hypertension and chronic kidney disease. Together, the examples demonstrate how different input profiles activate distinct rules, warnings, and decision-support outputs.

## 4. Discussion

### 4.1. Principal Findings

This study developed a transparent Python-based prototype that combines procedural dietetic rules, two meal-frequency pathways, exchange allocation, and menu-combination optimization. The two computational pathways should not be interpreted as having undergone equivalent forms of evaluation: exhaustive combinatorial analysis and mathematical optimization were applied only to the conventional multi-meal pathway, whereas the three-meal basal–bolus pathway was evaluated through predefined energy-specific exchange patterns and rule-based patient-specific decision logic. Its principal contribution is methodological: narrative recommendations were translated into explicit, inspectable conditions and quantitative constraints. The current implementation should be regarded as a transparent rule-based decision-support prototype intended to support professional decision making rather than to provide autonomous clinical recommendations.

Although the prototype was informed by Croatian guidance, international transferability cannot be assumed. Recommendations from professional organizations overlap in several broad principles but differ in emphasis, evidence grading, and individualization. The Croatian guidelines underlying the present prototype were developed on an evidence-based basis and incorporate recommendations from relevant international professional guidance, while also emphasizing individual and regional adaptation of nutritional therapy [15]. Adaptation to another regional setting would require a structured process comprising: (i) identification of the applicable local clinical nutrition guidelines; (ii) extraction and atomization of relevant recommendations into explicit computational rules; (iii) replacement or modification of energy, macronutrient, meal-pattern, exchange, and safety thresholds where these differ from the Croatian source guidelines; (iv) localization of food-composition data, exchange definitions, terminology, and candidate meals or recipes; and (v) technical verification and review by appropriately qualified local professionals before implementation. Any regionally adapted version would require separate validation rather than being assumed equivalent to the Croatian implementation.

For the evaluated 1800-kcal reference scenario, the exhaustive feasibility analysis provides additional insight into the selectivity of the proposed nutritional constraint framework. Of the 117,649 theoretically possible six-meal combinations generated from the available candidate set, only 5628 (4.78%) satisfied all nutritional constraints under the baseline ±6.5% energy tolerance. This finding indicates that the implemented nutritional constraints substantially narrow the combinatorial solution space while retaining multiple alternative feasible menu combinations. Sensitivity analysis showed that the feasible proportion increased from 2.71% at ±5% energy tolerance to 9.47% at ±10%, indicating that the selected energy-tolerance threshold materially affects the proportion of feasible combinations within the evaluated candidate space.

The objective-specific analysis further demonstrated trade-offs among the evaluated nutritional objectives within the available candidate space. Maximizing dietary fiber, for example, increased fiber intake by 51.4% compared with the minimum-carbohydrate solution but simultaneously increased carbohydrate intake by 16.9%. Conversely, minimizing carbohydrate intake reduced fiber by approximately 34.0% relative to the fiber-maximizing solution. The rule-based component first defines the feasible solution space according to the implemented nutritional constraints, after which the optimization layer selects among feasible menu combinations according to a specified nutritional objective. This approach preserves the predefined nutritional constraints while allowing flexibility in the selection of feasible menu combinations.

### 4.2. Comparison with Previous Studies

Computational approaches to nutrition decision support range from rule- and knowledge-based systems to mathematical optimization and, more recently, data-driven and generative artificial intelligence. In diabetes nutrition therapy, a systematic review by Spoladore et al. identified ontology-based recommender and decision-support systems as a relevant approach for representing expert knowledge and generating patient-tailored nutritional recommendations. The review also highlighted persistent challenges related to knowledge reuse, interoperability, and the integration of clinical expertise and guidelines into computable decision processes [27]. A subsequent implementation by Spoladore et al., the OnT2D-DSS, used an ontology-based knowledge representation to identify patient phenotypes and comorbidities and to provide personalized macro- and micronutrient recommendations for people with type 2 diabetes. Preliminary evaluation with nutrition experts and family physicians supported the correctness of its inferences and its potential usability in clinical practice [28]. These studies share with the present prototype an emphasis on explicit expert knowledge and human-interpretable decision support but differ in computational implementation and output: the present approach uses sequential procedural rules and lookup structures and extends the decision process from patient characteristics and meal-pattern selection to exchange allocation, nutrient-constrained menu selection, and optimization of complete daily meal combinations.

Mathematical optimization represents a complementary tradition in computerized diet planning. Mixed-integer linear programming has long been used to identify diets satisfying predefined nutritional requirements while optimizing a specified objective [29]. More recent work has expanded diet optimization by incorporating additional dimensions such as food compatibility and consumer acceptability; for example, van Wonderen et al. combined diet optimization with machine-learning methods to construct nutritionally adequate and acceptable menu plans [30]. The present prototype differs from approaches in which optimization constitutes the primary decision mechanism because optimization is applied only after the rule-based component has established the applicable meal pathway and nutritional constraints. Within the evaluated multi-meal pathway, MILP is additionally complemented by exhaustive enumeration of all 117,649 combinations generated from the 42-meal candidate dataset. This makes it possible not only to identify objective-specific optimal solutions but also to quantify the feasible solution space, examine sensitivity to the energy-tolerance threshold, and characterize trade-offs among carbohydrate, saturated-fat, fiber, and energy objectives. These analyses describe the behavior of the defined candidate space and should not be interpreted as evidence of globally optimal dietary solutions.

Recent AI-based approaches provide a further point of comparison. Conversational agents are increasingly being investigated in diabetes care, but a recent systematic review found substantial heterogeneity in their design and evaluation, with relatively few studies explicitly addressing safety and with generally limited methodological quality [31]. More specifically for dietary planning, Karakas et al. compared meal plans generated by large language models with a dietitian-designed, guideline-based reference diet for type 2 diabetes and reported systematic quantitative differences and inconsistent guideline adherence, including discrepancies in energy and fiber provision [32]. These findings illustrate an important distinction between generative dietary recommendations and deterministic, constraint-based decision support. The present prototype does not generate unrestricted dietary advice from probabilistic language output; instead, its recommendations are derived from inspectable rules, predefined exchange patterns, explicit nutritional constraints, and a bounded candidate meal dataset. Consequently, identical inputs within a fixed version of the rule base, dataset, and computational environment produce deterministic outputs, while missing nutrient information or infeasible combinations can be explicitly flagged rather than implicitly resolved through generative output.

This emphasis on transparency is also consistent with broader developments in decision-support methodology. Interpretability frameworks increasingly emphasize that decision-support outputs should allow users to understand the contribution of model inputs and the basis of computational recommendations [19]. At the same time, recent uncertainty-aware DSS research demonstrates the value of explicitly representing and propagating uncertainty rather than treating all input information as equally certain [33]. The present prototype does not implement Bayesian uncertainty propagation and therefore should not be considered an uncertainty-aware probabilistic DSS. Instead, uncertainty or incompleteness is handled operationally through explicit warnings and clinical-review flags when required information is unavailable. This represents a simpler deterministic strategy but preserves a visible distinction between outputs satisfying the implemented computational checks and those requiring additional professional assessment.

Taken together, comparison with previous studies suggests that the contribution of the present work does not lie in introducing rule-based decision support or mathematical diet optimization as independent concepts. Rather, its methodological contribution is the integration of explicit procedural dietetic rules, regimen-related meal-pattern selection, food-exchange allocation, nutrient-level feasibility constraints, MILP optimization, exhaustive combinatorial analysis, and traceable decision outputs within a single prototype for diabetic menu planning. This combination permits both patient-level rule execution and quantitative examination of the feasible menu space while retaining an inspectable computational pathway. However, unlike some previously evaluated knowledge-based systems, the present prototype has not yet undergone formal expert usability testing or prospective clinical validation; therefore, the current findings establish computational feasibility and methodological transparency rather than clinical effectiveness.

### 4.3. Clinical Implications and System Strengths

The use of regional foods and serving-based exchanges may facilitate future implementation in Croatian nutrition counselling. However, the illustrative food lists have not yet been evaluated for acceptability, cost, adherence, glycemic outcomes, or usability in clinical practice.

Strengths include explicit conditions, deterministic calculations, separation of the two computational pathways, and the ability to flag missing nutrient information. Reproducibility of the reported analyses currently depends on access to the version-specific 42-meal input file and the corresponding computational implementation. Clinical safety and efficacy have not been established and requires prospective evaluation.

### 4.4. Limitations

Several limitations should be considered when interpreting the present findings and planning further development. First, the current rule base is derived exclusively from clinical guidelines and does not incorporate individual patient preferences, cultural dietary habits, or specific food intolerances, meaning that real-world deployment still requires human customization. Second, the system focuses primarily on macronutrient adequacy, total caloric targets, sodium, and fiber; automated micronutrient auditing was not integrated into the validation module, which is particularly relevant for long-term therapeutic regimens. Third, the decision engine does not model real-time, dynamic glycemic responses, such as postprandial glucose variability, individualized insulin-to-carbohydrate ratios, or complex meal interaction effects. Fourth, although the system incorporates rule-based adjustments for physical activity, these adjustments rely on generalized clinical lookup tables rather than individualized exercise physiology or continuous glucose monitoring (CGM) data. Fifth, the system operates as a standalone decision-support tool and has not yet been embedded directly within Electronic Health Record platforms or evaluated within active clinical ward workflows, which may influence its practical usability and adoption. In addition, the sensitivity analysis was performed only for the 1800-kcal reference scenario. Because the composition and feasibility of meal combinations may differ across energy targets, the observed changes in feasible-solution proportions at ±5%, ±6.5%, and ±10% energy tolerances should not be assumed to apply directly to the other implemented energy levels (1200–2200 kcal). Energy-specific sensitivity analyses would be required to determine whether comparable feasibility patterns occur across these energy tiers.

Furthermore, the combinatorial and optimization analyses were restricted to the 42 candidate meals included in the computational dataset. Consequently, the reported feasible proportions and objective-specific optima characterize the available candidate space and should not be interpreted as global optima across all theoretically possible diabetic meal combinations. In addition, although rule extraction and computational encoding underwent multidisciplinary review within the author team, the implemented rule base has not yet undergone formal independent external validation by a separate panel of diabetes-specialist nutrition professionals or physicians.

### 4.5. Future Directions

The present prototype provides a methodological basis for further development of transparent computational decision-support tools in clinical nutrition. To build upon the foundation established in this study, future developments will follow several clear research avenues. A primary priority is the integration of automated micronutrient assessment and nutrient-density metrics to support more comprehensive evaluation of long-term therapeutic diets. Additionally, future development could evaluate extension of the rule base to concurrent chronic conditions, such as advanced renal disease, heart failure, and celiac disease. Future work could also explore a preference layer incorporating individual food preferences, cultural dietary habits, and local ingredient availability. Finally, future studies could evaluate the feasibility of integration with Electronic Health Record platforms and, following appropriate methodological development, with continuous glucose monitoring (CGM) data, as well as the future development and evaluation of patient-facing interfaces for dietetic education and self-management support, following appropriate usability, safety, and clinical validation.

A further technical feature of the present implementation is the integration of an interactive patient-input and recommendation layer. Unlike a static menu calculator, the prototype provides a traceable computational pathway from patient characteristics to activated rules, nutritional allocation, validation status, clinical warnings, and resulting recommendations. A further potential extension would be adaptation of the rule base for individuals with prediabetes or insulin resistance who do not meet diagnostic criteria for diabetes. Lifestyle interventions, including individualized dietary modification, have an established role in preventing or delaying progression to type 2 diabetes in individuals at increased risk [34]. However, this population is outside the scope of the present prototype; therefore, extension to this group would require population-specific nutritional rules, appropriate clinical thresholds, and separate validation before implementation. This traceable workflow enables the clinician to distinguish between outputs satisfying the implemented computational checks and outputs requiring additional professional assessment, thereby reinforcing the role of the prototype as a decision-support tool rather than an autonomous clinical decision maker. The intended long-term use of the prototype is as a transparent, professionally supervised decision-support tool for diabetes nutrition care. Following further software verification, usability assessment, and clinical validation, the authors intend to make the developed tool openly accessible to facilitate its use, independent evaluation, reproducibility, and further development. At the present stage, no specific institutional deployment pathway has been established, and implementation within national healthcare systems, universities, or other healthcare settings would require separate evaluation and appropriate institutional arrangements.

## 5. Conclusions

This study presents a transparent Python-based decision-support prototype for diabetic menu planning. The system combines procedural therapy- and meal-frequency rules, exchange allocation, nutritional checks, and mathematical optimization of candidate meal combinations. It distinguishes a conventional multi-meal pattern from a three-meal basal–bolus pattern and makes the assumptions and limitations of each pathway explicit.

The findings demonstrate computational feasibility within the available datasets and tested scenarios, but they do not establish clinical effectiveness. All optimization results and feasible-solution proportions reported in this study are conditional on the current 42-meal candidate dataset and should not be directly generalized to other meal libraries or clinical contexts without further evaluation. Further development should include independent software testing, expert usability assessment, and prospective clinical validation before consideration for routine clinical use. Accordingly, the current prototype should be regarded as a professional decision-support tool for use by qualified dietitians/nutritionists and other appropriately qualified healthcare professionals, rather than as a replacement for professional dietary assessment or an autonomous patient-facing system.

Future development should focus on a unified version-controlled food and recipe database, reproducible packaging of code and input data, patient-preference and cost constraints, and evaluation with qualified dietitians/nutritionists and people living with diabetes.

## Figures and Tables

**Figure 1:**
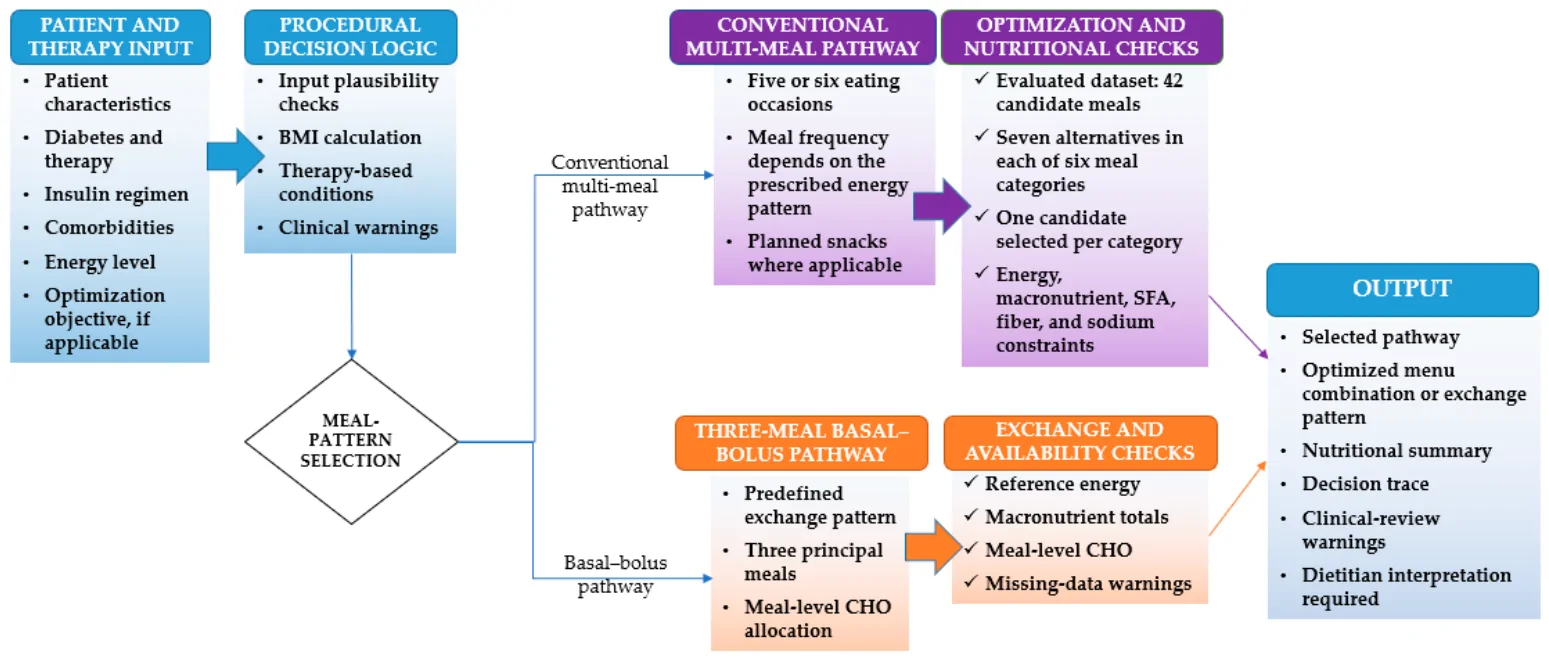
Conceptual architecture of the Python-based decision-support prototype for diabetic menu planning. Patient and therapy inputs are evaluated using explicit procedural conditions to select either the conventional multi-meal pathway or the three-meal basal–bolus pathway. The conventional pathway applies binary linear optimization to a structured candidate-meal dataset, whereas the basal–bolus pathway retrieves a predefined energy-specific exchange pattern. Nutritional checks are limited to variables available in the corresponding dataset, and unavailable clinically relevant information triggers a professional-review warning.

**Figure 2:**
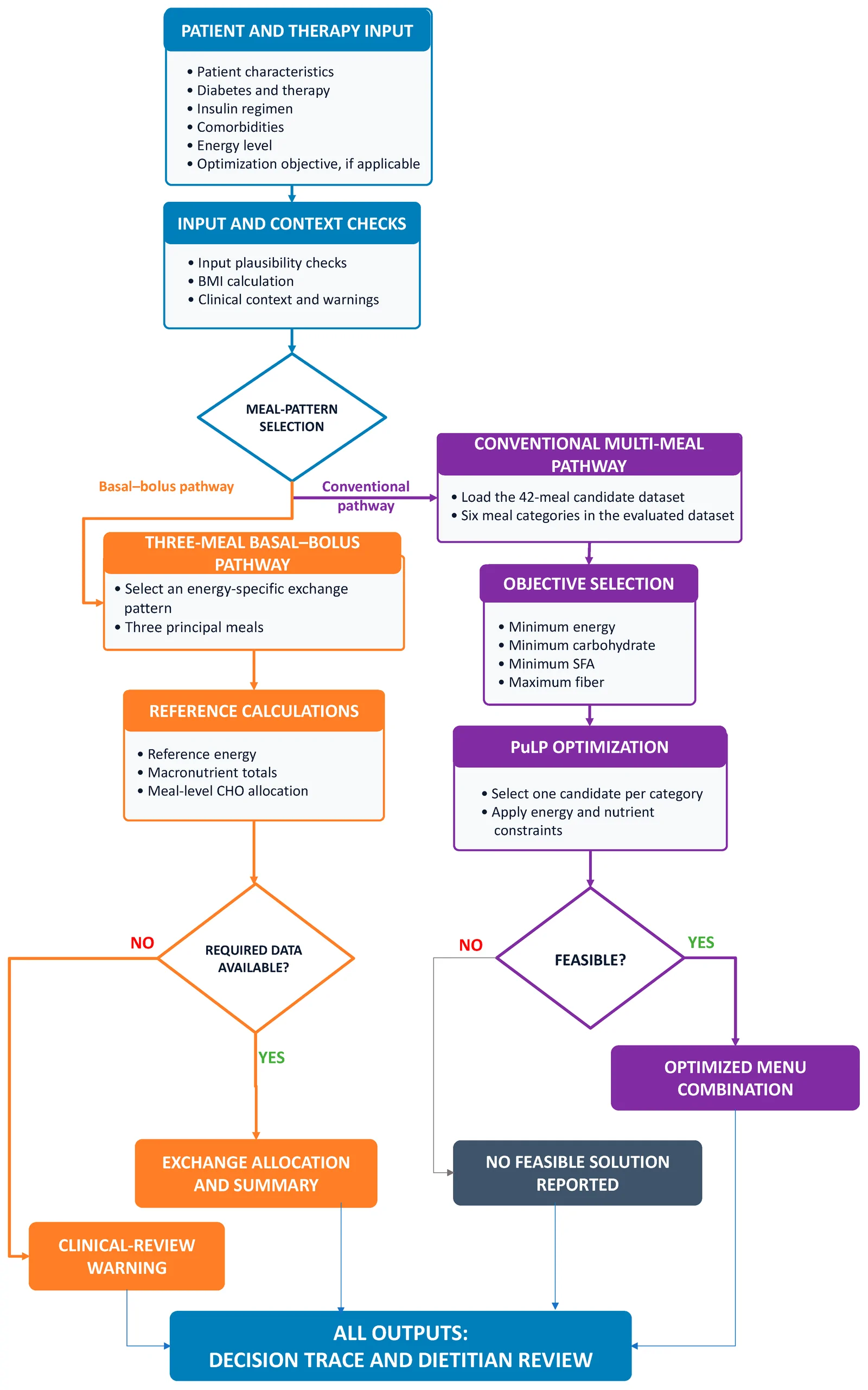
Implemented computational workflow of the diabetic menu-planning prototype. Following input plausibility checks and assessment of the therapeutic regimen, the program selects either the conventional multi-meal optimization pathway or the three-meal basal–bolus exchange pathway. The conventional pathway uses PuLP to select one candidate from each of multi-meal categories under predefined nutritional constraints and reports whether a feasible solution was identified. The basal–bolus pathway retrieves an energy-specific exchange pattern and reports reference energy, macronutrient totals, meal-level carbohydrate allocation, and clinical-review warnings for unavailable nutrient information. Both pathways produce a traceable output requiring professional interpretation.

**Figure 3:**
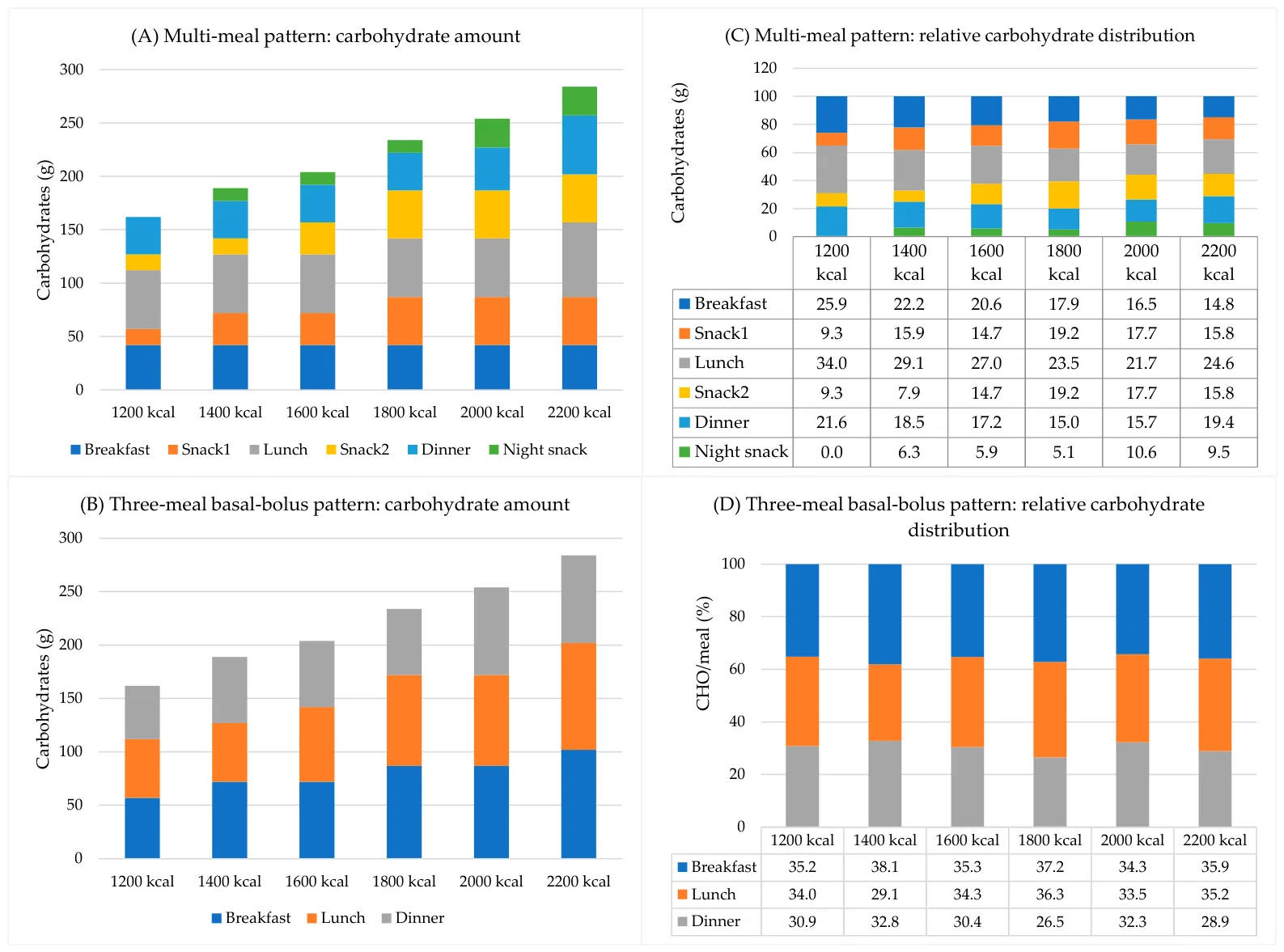
Carbohydrate allocation across prescribed energy levels for the (**A**) conventional multi-meal pattern and (**B**) three-meal basal–bolus pattern, with the corresponding relative carbohydrate distribution shown in (**C**,**D**), respectively.

**Figure 4:**
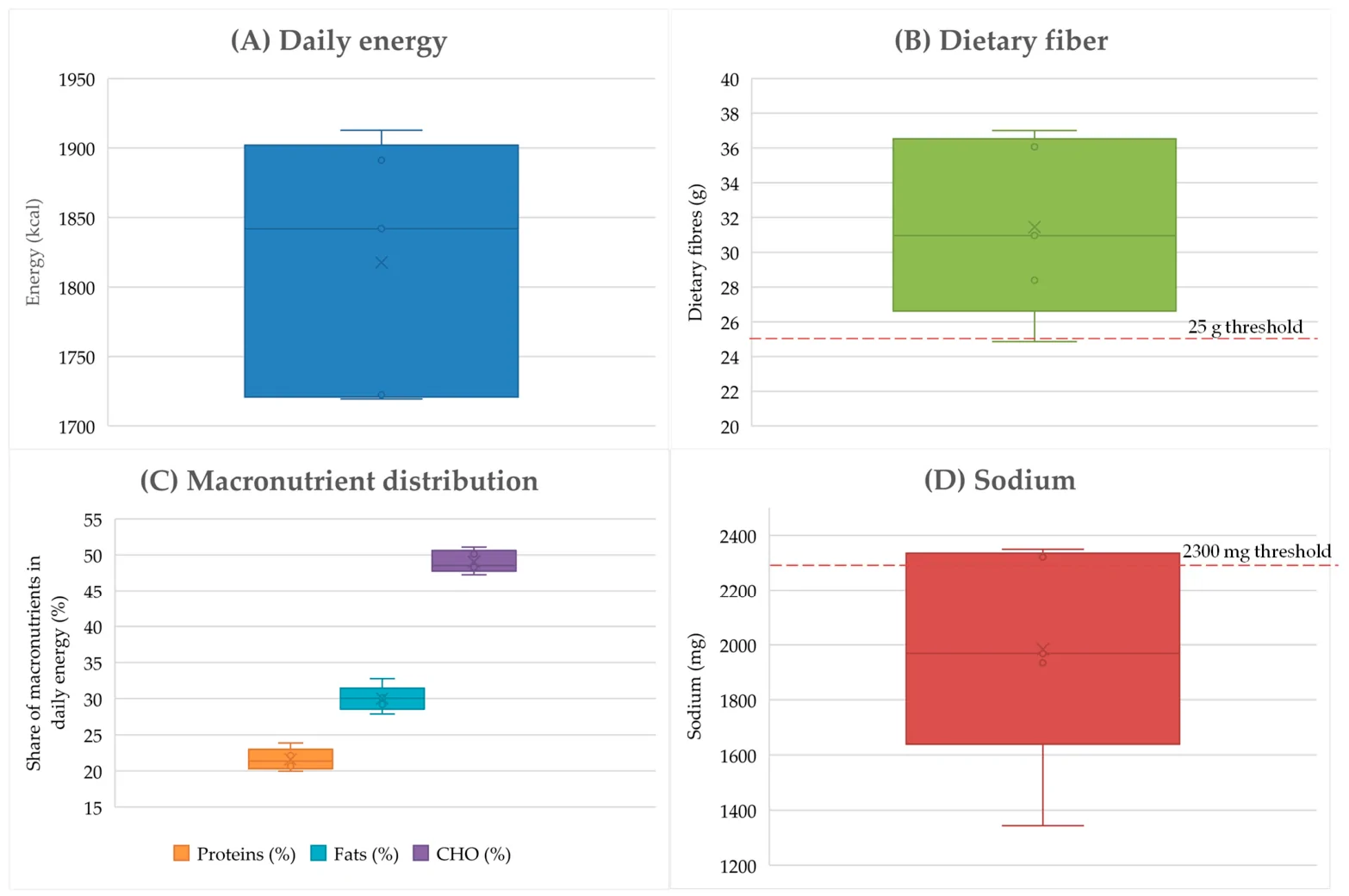
Nutritional variability across five selected multi-meal daily menus from the nominal 1800-kcal candidate set: (**A**) energy; (**B**) dietary fiber; (**C**) macronutrient contribution to energy; and (**D**) sodium. Box plots show the median, interquartile range, mean (X), individual observations, and min–max values. Dashed lines denote the 25 g fiber and 2300 mg sodium analysis thresholds. One menu was slightly below the fiber threshold and two slightly exceeded the sodium threshold.

**Table 1:** Knowledge sources integrated into the system.

Knowledge Source	Purpose in Decision-Support Prototype	Core Data/Output
Croatian MNT Guidelines (2023)	Clinical rules and safety limits	Energy equations, macronutrient ranges, clinical targets
Exchange Lists System	Portion and exchange allocation	Standardized exchange equivalents (CHO, protein, fat)
Food Composition Database	Exact nutrient calculation	Energy, macronutrient, fiber, and sodium values per 100 g
Hospital Recipes Standard	Practical menu generation	Standardized preparation rules and culinary combinations

**Table 2:** Modular structure of the rule base.

Module	Function	Representative Logic
Input checks	Checks plausibility and required variables	IF age, mass, or height is implausible, THEN request correction
BMI and context	Calculates BMI and adds contextual advice	IF BMI ≥ 25 kg/m^2^, THEN flag individualized energy review
Meal-pattern selection	Selects a conventional five- or six-meal pathway or a three-meal basal–bolus pathway	IF modern basal–bolus regimen, THEN three principal meals may be selected
Exchange allocation	Retrieves energy-specific serving pattern	IF three-meal pathway AND a supported target energy level is selected, THEN retrieve the corresponding energy-specific exchange pattern
Optimization	Selects one candidate per six-meal category	Minimize selected objective subject to nutrient constraints
Validation/warnings	Evaluates available variables	IF sodium is unavailable, THEN clinical review required

**Table 3:** Rule-Based differences between the conventional multi-meal and three-meal basal–bolus patterns.

Characteristic	Conventional Five to Six-Meal Pattern	Three-Meal Basal–Bolus Pattern
Therapeutic context	Lifestyle/oral therapy, basal insulin, or regimens for which snacks are planned	Modern basal–bolus or multiple-daily-injection regimen when clinically appropriate
Eating occasions	Three main meals plus planned snacks	Three principal meals; routine snacks generally not required
Carbohydrate timing	Distributed across main meals and snacks	Concentrated in three meals and coordinated with rapid-acting insulin
Food quality	Individualized, fiber-rich pattern where appropriate	Same food-quality principles; no automatic fiber or texture restriction
Clinical caveat	Adapt to medication and hypoglycemia risk	Adapt to insulin-to-carbohydrate ratio, activity, and hypoglycemia risk

**Table 4:** Illustrative multi-meal and three-meal basal–bolus structures.

Eating Occasion	Conventional Multi-Meal Pattern	Three-Meal Basal–Bolus Pattern
Breakfast	Included	Included; carbohydrate coordinated with mealtime insulin
Morning snack	Planned according to regimen	Not routinely required
Lunch	Included	Included; carbohydrate coordinated with mealtime insulin
Afternoon snack	Planned according to regimen	Not routinely required
Dinner	Included	Included; carbohydrate coordinated with mealtime insulin
Night snack	Planned when clinically indicated	Not routinely required; individualized for hypoglycemia risk

**Table 5:** Optimization performance and nutritional characteristics of feasible daily menu solutions.

Optimization Objective	Energy (kcal)	Protein (g)	Fat (g)	CHO (g)	SFA (g)	Fiber (g)	Sodium (mg)	Status
Min Energy	1776.97	86.75	52.43	239.52	13.75	35.50	1097.67	PASS
Min CHO	1796.14	109.62	57.39	210.30	13.63	27.35	1996.19	PASS
Min SFAs	1779.27	102.30	54.78	219.25	12.49	33.33	1927.09	PASS
Max Fiber	1795.07	90.66	56.53	245.94	13.76	41.42	1534.05	PASS

CHO, carbohydrates; SFAs, saturated fatty acids. PASS indicates compliance with all implemented optimization constraints.

**Table 6:** Sensitivity of the feasible solution space and objective-specific optima to alternative energy-tolerance thresholds for the 1800-kcal reference scenario. All non-energy nutritional constraints were held constant.

Energy Tolerance	Acceptable Energy Range (kcal/Day)	Feasible Combinations (n)	Feasible Combinations (%)	Min Energy (kcal)	Min CHO (g)	Min SFA (g)	Max Fiber (g)
±5%	1710–1890	3192	2.71	1807.37	213.99	12.52	39.01
±6.5%	1683–1917	5628	4.78	1776.97	210.3	12.49	41.42
±10%	1620–1980	11,144	9.47	1710.13	203.66	10.39	41.42

**Table 7:** Selected trade-offs between objective-specific optimal solutions under the baseline ±6.5% energy tolerance.

Comparison	Primary Benefit	Associated Trade-Off
Max Fiber vs. Min CHO	Fiber: 27.35 → 41.42 g (+51.4%)	CHO: 210.30 → 245.94 g (+16.9%)
Min CHO vs. Max Fiber	CHO reduced by 14.5% relative to Max Fiber	Fiber reduced by 34.0%
Min SFA vs. Max Fiber	SFA: 13.76 → 12.49 g (−9.2%)	Fiber: 41.42 → 33.33 g (−19.5%)

**Table 8:** Representative patient-specific inference trace, nutritional validation, and decision output of the interactive decision-support prototype.

Component	Result
Patient profile	Male, 40 years, Type 1 diabetes
BMI	27.70 kg/m^2^
Therapy	Modern basal–bolus insulin regimen; pen administration
Comorbidity	Hypertension; no CKD
Selected dietary branch	Three-meal basal–bolus pattern
Selected energy pattern	1600 kcal
Activated rules	R01, R11, R20, R40, R41, R42
CHO	189 g—exact reference match
Protein	78 g—exact reference match
Fat	38.5 g—exact reference match
Reference energy	1527.5 kcal
CHO distribution	Breakfast 72 g; Lunch 55 g; Dinner 62 g
Clinical warning	Sodium requires separate assessment
Final status	P ASS WITH CLINICAL REVIEW REQUIRED

**Figure A1:**
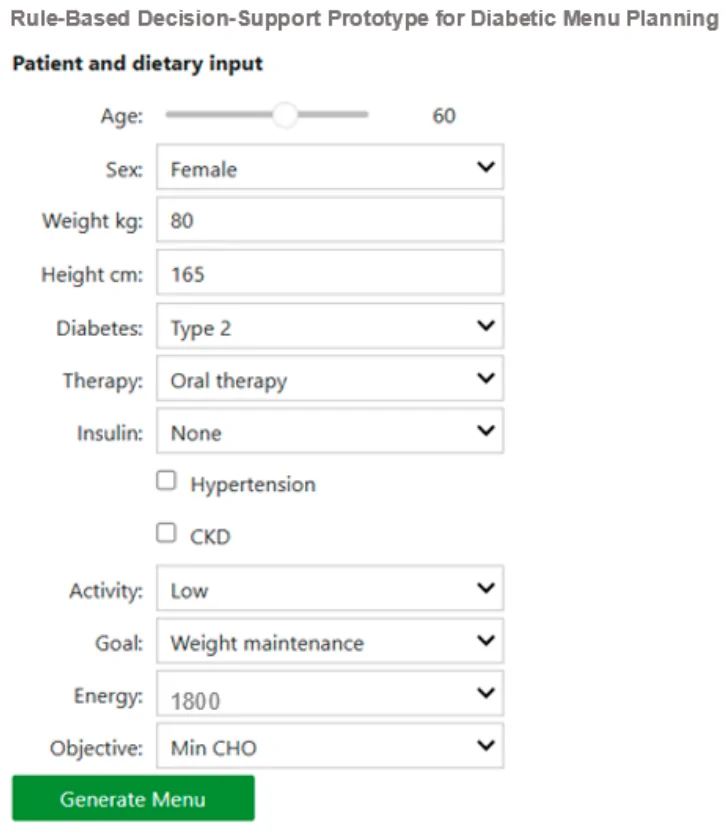
Interactive patient-input interface of the rule-based decision-support prototype for diabetic menu planning.

## Data Availability

The computational dataset and supporting materials underlying the findings of this study are available from the corresponding author upon reasonable request. The complete source code and implementation notebook are not currently deposited in a public repository because options for intellectual-property protection and subsequent dissemination of the developed prototype are under consideration. Sufficient methodological details, including the optimization formulation, solver configuration, nutritional constraints, and computational procedures, are provided in the manuscript to support evaluation of the reported methodology.

## Supplementary Materials

The following supporting information can be downloaded at: https://www.mdpi.com/article/10.3390/nu18193140/s1, Table S1: Daily Exchange Serving Targets Across All Energy Levels (1200–2200 kcal); Table S2: Structured Meal-by-Meal Exchange Allocation Protocol for the Conventional Multi-Meal Pattern.

## Abbreviations

The following abbreviations are used in this manuscript:

AI	Artificial intelligence
BMI	Body mass index
CHO	Carbohydrate
CKD	Chronic kidney disease
DM	Diabetes mellitus
EHR	Electronic health record
GI	Glycemic index
MNT	Medical nutrition therapy
MILP	Mixed-integer linear programming
SFA	Saturated fatty acids

## Appendix A

### Appendix A.1. Interactive Patient-Input Interface and Operational Characteristics

**Table A1:** Operational characteristics of the decision-support prototype.

Characteristic	Outcome
Inference mechanism	Sequential procedural rules
Knowledge representation	Explicit Python conditions and lookup tables
Rule execution	Deterministic
Menu generation	Optimization or retrieval of predefined exchange patterns
Validation	Variable-specific checks and warnings
Rule conflicts	None detected
Explainability	Printed decision trace
Reproducibility	Dependent on code and version-matched input data

### Appendix A.2. Additional Representative Decision-Trace Outputs

To further illustrate the traceability of the implemented decision logic across different patient and clinical scenarios, three additional representative decision-trace outputs are presented below. The examples demonstrate differences in pathway selection, activated rules, optimization or exchange-allocation outputs, clinical warnings, and resulting recommendations.

**Table A2:** Representative decision-trace output for the conventional multi-meal optimization pathway.

Component	Result
Patient profile	Female, 50 years, Type 2 diabetes
BMI	22.04 kg/m^2^ (60 kg; 165 cm)
Therapy	Oral therapy
Comorbidity	No hypertension; no CKD
Selected dietary branch	Conventional multi-meal optimization pathway
Reference energy scenario	1800 kcal
Optimization objective	Minimize carbohydrate intake
Activated rules	R02: Standard diabetic regimen activated; R12: BMI < 25 → standard weight-status rule
Solver/optimization status	Optimal
Selected meal combination	B3 + Sn1_1 + L1 + Sn2_6 + D1 + N_Sn1
Energy	1796.14 kcal
Protein	109.62 g
Fat	57.39 g
CHO	210.30 g
SFA	13.63 g
Fiber	27.35 g
Sodium	1996.19 mg
Constraint evaluation	All implemented nutritional constraints satisfied
Clinical warning	None
Recommendation	The selected menu minimizes carbohydrate intake while satisfying all active nutritional constraints; professional interpretation remains required.
Final status	PASS

**Table A3:** Representative decision-trace output for the three-meal PEN/basal–bolus pathway with chronic kidney disease.

Component	Result
Patient profile	Male, 58 years, Type 2 diabetes
BMI	31.14 kg/m^2^ (90 kg; 170 cm)
Therapy	Insulin + oral therapy; PEN administration
Comorbidity	CKD; no hypertension
Selected dietary branch	Three-meal PEN/basal–bolus pathway
Selected energy pattern	1900 kcal
Activated rules	R01: PEN insulin delivery → PEN regimen activated; R10: BMI ≥ 30 → obesity-related energy review; R30: CKD → individualized protein review required; R40: PEN regimen → PEN exchange allocation activated; R41: Target energy 1900 kcal → 1900-kcal PEN pattern selected; R42: PEN regimen → three-main-meal distribution activated
CHO	234 g—exact exchange/reference match
Protein	95 g—exact exchange/reference match
Fat	46.5 g—exact exchange/reference match
Reference energy	1791.05 kcal
CHO distribution	Breakfast 87 g; Lunch 85 g; Dinner 62 g
Clinical warning	CKD identified; protein intake requires individualized renal dietitian and clinical assessment
Recommendations	Individualized energy-intake and weight-management review; follow the 1900-kcal PEN exchange pattern; maintain meal-specific carbohydrate distribution; individualized renal assessment of protein intake
Final status	P ASS WITH CLINICAL REVIEW REQUIRED

**Table A4:** Representative decision-trace output for the three-meal PEN/basal–bolus pathway with hypertension and chronic kidney disease.

Component	Result
Patient profile	Female, 67 years, Type 2 diabetes
BMI	27.34 kg/m^2^ (70 kg; 160 cm)
Therapy	Insulin + oral therapy; PEN administration
Comorbidity	Hypertension and CKD
Selected dietary branch	Three-meal PEN/basal–bolus pathway
Selected energy pattern	2100 kcal
Activated rules	R01: PEN insulin delivery → PEN regimen activated; R11: BMI 25–29.9 → overweight-related energy review; R20: Hypertension → sodium-related dietary constraint; R30: CKD → individualized protein review required; R40: PEN regimen → PEN exchange allocation activated; R41: Target energy 2100 kcal → 2100-kcal PEN pattern selected; R42: PEN regimen → three-main-meal distribution activated
CHO	254 g—exact exchange/reference match
Protein	107 g—exact exchange/reference match
Fat	53.0 g—exact exchange/reference match
Reference energy	1983.80 kcal
CHO distribution	Breakfast 87 g; Lunch 85 g; Dinner 82 g
Clinical warnings	Sodium cannot be fully quantified from the current PEN exchange dataset and requires separate dietitian verification; CKD requires individualized assessment of protein intake
Recommendations	Weight-management and energy-intake review; lower-sodium food selection with separate sodium verification; individualized renal assessment of protein intake; follow the 2100-kcal PEN exchange pattern and maintain meal-specific carbohydrate distribution
Final status	PASS WITH CLINICAL REVIEW REQUIRED

### Appendix A.3. Structured Scenario Matrix

To provide a structured overview of the representative scenarios presented in the manuscript and Appendix A, Table A5 summarizes variation in diabetes type, treatment regimen, BMI category, comorbidity profile, energy level, computational pathway, and resulting rule activation. The matrix is intended as a descriptive summary of scenario coverage rather than as a formal software-validation test suite.

**Table A5:** Structured scenario matrix for functional demonstration of the decision-support prototype.

Scenario	Diabetes/Therapy	BMI Category	HTN	CKD	Energy	Pathway	Main Demonstrated Behavior
Primary example (Table 8)	T1D/modern basal–bolus	Overweight	Yes	No	1600 kcal	Three-meal	Basal–bolus selection; HTN warning
Appendix scenario 1	T2D/oral therapy	Normal weight	No	No	1800 kcal	Conventional	MILP optimization; no comorbidity warning
Appendix scenario 2	T2D/basal–bolus PEN	Obesity	No	Yes	1900 kcal	Three-meal	CKD clinical-review warning
Appendix scenario 3	T2D/basal–bolus PEN	Overweight	Yes	Yes	2100 kcal	Three-meal	Combined HTN/CKD warnings
