# Supplementary material for: A Transparent Rule-Based Decision-Support Prototype for Diabetic Menu Planning and Meal-Combination Optimization: Development and Computational Evaluation
Source: Nutrients. 2026 Sep 23;18(19):3140. doi: 10.3390/nu18193140 (PMC13666910; doi:10.3390/nu18193140)
Supplement: Supplementary file 1 [file nutrients-18-03140-s001.zip › nutrients-4569373-supplementary.pdf]

nutrients

MDPI

Supplementary Material: Comprehensive Exchange & Meal Allocation Tables

The exchange targets and meal-level allocations presented in Tables S1 and S2 were constructed using a food-exchange framework in which foods are grouped according to comparable macronutrient and energy contributions [23]. For computational interpretation in the present prototype, one carbohydrate exchange was treated as approximately 15 g carbohydrate, one protein exchange as approximately 7 g protein, one fat exchange as approximately 5 g fat, and one milk/yogurt exchange as approximately 12 g carbohydrate and 8 g protein [23]. Energy-specific exchange patterns were subsequently distributed across the prescribed eating occasions according to the corresponding meal pattern. The resulting values in Tables S1 and S2 represent operational exchange-based planning allocations used in the prototype rather than direct nutrient-composition calculations; exact energy and nutrient totals depend on the foods selected within each exchange group.

Table S1. Daily Exchange Serving Targets Across All Energy Levels (1200–2200 kcal)

Recommended Energy (kcal)	Servings					
	Carbohydrate	Protein	Non-Starchy Vegetables	Fruit	Milk/Yogurt	Added Fats/Oils
1200	10–11	5	3–4	1	1	2
1400	12–13	6	3–5	1–2	1	3
1600	13-14	6	5-6	2	1-2	3
1800	14–15	7	4–6	2	1–2	3–4
2000	16–17	7–8	4–6	2	2	4
2200	18–19	8–9	5–7	2–3	2	5

Serving ranges indicate allowable variation within the exchange-based planning pattern and are intended to support individualized food selection within the corresponding food groups.

Table S2. Structured Meal-by-Meal Exchange Allocation Protocol for the Conventional Multi-Meal Pattern

Meal	Food group and substitutes	1200 kcal	1400 kcal	1600 kcal	1800 kcal	2000 kcal	2200 kcal
Breakfast	Milk	1	1	1	1	1	1
	Bread	2	2	2	2	2	2
	Meat	1	1	1	2	2	2
	Fats	0	0	0	1	1	1
Snack 1	Bread	0	1	1	1	1	1
	Meat	0	0	0	0	1	1
	Fruit	1	1	1	2	2	2
	Fats	0	0	0	0	0	1
Lunch	Vegetable	2	2	2	2	2	2
	Bread	2	2	2	2	2	3
	Meat	2	2	2	2	2	2
	Fats	2	2	2	2	2	2
	Fruit	1	1	1	1	1	1
Snack 2	Bread	0	0	1	1	1	1
	Meat	0	0	1	1	1	1
	Fats	0	0	0	0	1	1
	Fruit	1	1	1	2	2	2
Dinner	Vegetable	1	1	1	1	2	2
	Bread	2	2	2	2	2	3
	Meat	2	2	2	2	2	2
	Fats	2	2	2	2	2	2
	Fruit	0	0	0	0	0	0

Night snack	Milk	0	1	1	1	1	1
	Bread	0	0	0	0	1	1
	Meat	0	0	0	0	0	0

Note: "Bread" and "Meat" denote food-exchange groups rather than mandatory individual foods. Foods within each exchange group may be substituted with nutritionally equivalent alternatives according to the exchange-list framework [23].
